# Simultaneous integrated boost intensity-modulated radiotherapy (SIB-IMRT) combined with nimotuzumab for locally advanced esophageal squamous cell carcinoma (ESCC): A phase II clinical trial

**DOI:** 10.1186/s12885-024-12427-y

**Published:** 2024-06-04

**Authors:** Lan Wang, Lihong Liu, Yu Cao, Xiaoxi Chen, Shutang Liu, Xiaoning Li, Jing Han, Qi Wang, Chun Han

**Affiliations:** 1 Department of Radiation Oncology, The Fourth Hospital of Hebei Medical University, Shijiazhuang, 050011 China; 2 Anti-Cancer Association of Hebei Province, The Fourth Hospital of Hebei Medical University, Shijiazhuang, China; 3 Department of Medical Oncology, The Fourth Hospital of Hebei Medical University, Shijiazhuang, China

**Keywords:** Esophageal squamous cell cancer, SIB-IMRT, Nimotuzumab, Safety, Efficacy

## Abstract

**Objective:**

To evaluate the feasibility, safety and efficacy of concurrent simultaneous integrated boost intensity-modulated radiotherapy (SIB-IMRT) combined with nimotuzumab in the treatment of locally advanced esophageal squamous cell cancer (ESCC).

**Methods:**

Eligible patients were histologically proven to have locally advanced ESCC, and were unable to tolerate or refuse concurrent chemoradiotherapy (CCRT). Enrolled patients underwent concurrent SIB-IMRT in combination with nimotuzumab. SIB-IMRT: For the planning target volume of clinical target volume (PTV_-C_), the prescription dose was 50.4 Gy/28fractions, 1.8 Gy/fraction, 5fractions/week, concurrently, the planning target volume of gross tumor (PTV_-G_) undergone an integrated boost therapy, with a prescription dose of 63 Gy/28fractions, 2.25 Gy/fraction, 5 fractions/week. Nimotuzumab was administered concurrently with radiotherapy, 200 mg/time, on D1, 8, 15, 22, 29, and 36, with a total accumulation of 1200 mg through intravenous infusion. The primary endpoint of the study was the safety and efficacy of the combined treatment regimen, and the secondary endpoints were 1-year, 2-year, and 3-year local control and survival outcomes.

**Results:**

(1) From December 2018 to August 2021, 35 patients with stage II-IVA ESCC were enrolled and 34 patients completed the full course of radiotherapy and the intravenous infusion of full-dose nimotuzumab. The overall completion rate of the protocol was 97.1%. (2) No grade 4–5 adverse events occurred in the entire group. The most common treatment-related toxicity was acute radiation esophagitis, with a total incidence of 68.6% (24/35). The incidence of grade 2 and 3 acute esophagitis was 25.7% (9/35) and 17.1% (6/35), respectively. The incidence of acute radiation pneumonitis was 8.6% (3/35), including one case each of Grades 1, 2, and 3 pneumonitis. Adverse events in other systems included decreased blood cells, hypoalbuminemia, electrolyte disturbances, and skin rash. Among these patients, five experienced grade 3 electrolyte disturbances during the treatment period (three with grade 3 hyponatremia and two with grade 3 hypokalemia). (3) Efficacy: The overall CR rate was 22.8%, PR rate was 71.4%, ORR rate was 94.2%, and DCR rate was 97.1%.(4) Local control and survival: The 1-, 2-, and 3-year local control (LC) rate, progression-free survival(PFS) rate, and overall survival(OS) rate for the entire group were 85.5%, 75.4%, and 64.9%; 65.7%, 54.1%, and 49.6%; and 77.1%, 62.9%, and 54.5%, respectively.

**Conclusions:**

The combination of SIB-IMRT and nimotuzumab for locally advanced esophageal cancer demonstrated good feasibility, safety and efficacy. It offered potential benefits in local control and survival. Acute radiation esophagitis was the primary treatment-related toxicity, which is clinically manageable. This comprehensive treatment approach is worthy of further clinical exploration (ChiCTR1900027936).

## Introduction

For locally advanced unresectable esophageal cancer, curative concurrent chemoradiotherapy (CCRT) is the standard treatment modality [1–3]. Over the years, with the continuous improvement of radiotherapy technology and equipment, as well as chemotherapy drugs, the efficacy data of CCRT for esophageal cancer has been continuously improved, and its 5-year overall survival rate has increased from the previously reported 26% [2] to 39.9% [4]. In the era of immunotherapy, although multiple studies [5–9] are ongoing on the combination of chemoradiotherapy and immunotherapy for locally advanced esophageal cancer, based on the existing results, CCRT remains the most recommended treatment modality. However, in real-world studies [10], due to various reasons such as old age, poor performance status, malnutrition, comorbidities, and technical equipment limitations of treatment institutions, the proportion of patients who can receive CCRT is not high (45.5%). The remaining majority of patients will primarily receive sequential chemoradiotherapy, radiotherapy alone, or more individualized treatment regimens, resulting in a subsequent decline in efficacy data. For patients with esophageal cancer who cannot tolerate or refuse CCRT, identifying a more effective and less toxic comprehensive treatment regimen to bring therapeutic benefits to a larger patient population, it is an urgent clinical issue to be addressed. Based on the aforementioned background, considering the local regional control advantage of the SIB-IMRT technique [11] and the high safety profile demonstrated by nimotuzumab in esophageal cancer studies [12, 13], we designed this phase II clinical trial. The primary endpoint of the study is to evaluate the feasibility, safety, and efficacy of the new combined approach of SIB-IMRT and nimotuzumab in treating locally advanced esophageal cancer. The secondary endpoint is the local control and survival outcomes of the patients.

## Materials and methods

### Study design

This study is a prospective, single-arm, phase II clinical trial. The study was conducted in accordance with the Declaration of Helsinki. All patients gave their written informed consent. The fourth hospital of Hebei medical university ethics committees approved the protocol (reference: 2,019,040). The clinical trial registration number is ChiCTR1900027936(Date of registration: December 6, 2019).

### Patients and eligibility criteria

The inclusion criteria were as follows [14]: ⑴ ESCC confirmed by histopathology; ⑵ Locally advanced ESCC according to baseline assessment (Thoracic and abdominal CT, ultrasound of lymph nodes in the neck vascular and supraclavicular regions, esophageal barium meal imaging, gastroscopy or endoscopic ultrasound, if necessary, further assessment such as thoracic MRI, PET-CT, ECT, etc., can be made). Stage cT_1~2_N + M_0_ or cT_3-4_N_0~3_M_0_ disease according to the 8th American Joint Committee on Cancer TNM staging system was enrolled; ⑶ Patients were inoperable, could not tolerate or refuse CCRT; ⑷ ECOG PS 0–2; ⑸ Voluntary written consent provided prior to treatment.

The exclusion criteria were as follows: ⑴ Esophagobronchial or esophagomediastinal fistula; ⑵ Serious heart, liver, and/or kidney insufficiency; ⑶ Serious infectious diseases; ⑷ Relapse disease or distant metastasis; ⑸ Previous diagnosed malignant disease.

### Treatment

**Fig. 1:**
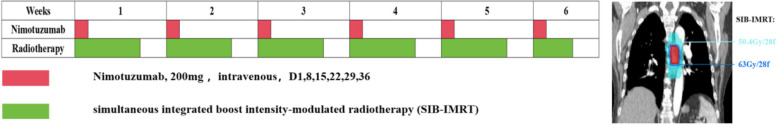
Treatment procedure

All patients received concurrent SIB-IMRT combined with nimotuzumab in intravenous infusion. The treatment procedure is shown in Fig. 1.

### Radiotherapy

All patients underwent computed tomography (CT)-based treatment simulation in the supine position, and 3-mm thick images were obtained throughout the entire neck, thorax, and upper abdomen. The scanned images were transferred to a three-dimensional (3D) planning system. The GTV, CTV, PTV, and normal organs at risk (OAR) were delineated layer by layer. The GTV included primary tumors (GTV_-P_) and lymph node metastasis (GTV_-n_). The GTV_-P_ included all tumors that were found using a CT scan, esophageal barium, endoscopy/endoscopic ultrasonography (EUS), and PET-CT. The GTV_-n_ was defined as any lymph node diagnosed as or highly suspected of being metastatic. The CTV of a primary tumor (CTV_-P_) was defined as the GTV_-P_ plus a 2-cm margin superiorly and inferiorly and a 0.5-cm margin laterally along the esophagus. For the CTV of the lymph node (CTV_-n_), involved-field radiotherapy (IFI) was used for the majority of patients. However, if the primary tumor was in the cervical or upper thoracic esophagus, the CTV_-n_ encompassed the elective nodal area including the bilateral supraclavicular and upper mediastinal lymph node regions. The PTV of the clinical target volume (PTV_-C_) was generated by adding a 1-cm margin craniocaudally, a 0.5-cm margin laterally along the CTV_-P_, and a uniform 0.5-cm margin around CTV-n. The PTV_-G_ was defined using the GTV (GTV_-P_ + GTV_-n_) plus a 0.3–0.5 cm margin. In this study, the PTV_-G_ and PTV_-C_ received two prescription doses of 63 Gy and 50.4 Gy simultaneously. The lower dose was delivered to the PTV_-C_ (50.4 Gy/28 fractions, 1.8 Gy per fraction), and the higher dose was escalated to the PTV_-G_ (63 Gy/28 fractions, 2.25 Gy per fraction). A prescription dose was defined as 95% of the receiving dose of the PTV, with the difference of the internal target dose uniformity of < 5%, and the internal target maximum dose point of ≤ 110%. The OAR included the spinal cord, lungs, and the heart. The treatment plan generally required the entire lungs V5 ≤ 55 – 60%, V20 ≤ 25 – 30%, and V30 ≤ 18%; a mean heart dose of ≤ 26 – 30 Gy; and a maximum spinal cord dose of < 45 Gy. The representative SIB-IMRT planning images with contours are shown in Fig. 1.

### Targeted therapy

The patients received weekly infusions of 200 mg of nimotuzumab diluted in 250 ml of normal saline concurrently with SIB-IMRT for 6 weeks (accumulated dose: 1200 mg).

### Endpoints

The primary observational endpoint was the safety and efficacy of SIB-IMRT combined with nimotuzumab in the treatment of locally advanced ESCC. The specific indicators included treatment completion rate, incidence and severity of treatment-related toxic side effects (rash, nausea/vomiting, bone marrow suppression, liver and kidney toxicity reactions, radiation-induced esophageal and pulmonary injury, bleeding, etc.), as well as short-term therapeutic effects. The secondary endpoints were 1-, 2-, and 3-year local control (LC) rates, progression-free survival (PFS) rates, and overall survival (OS) rates.

### Criteria for evaluation of toxicities and therapeutic effects

The National Cancer Institute Common Terminology Criteria for Adverse Events version 4.03(CTCAE V4.03) was used to assess toxicities. The Response Evaluation Criteria in Solid Tumors (version 1.1) [15] were used to evaluate treatment response. The comprehensive evaluation of therapeutic effect was mainly based on imaging results such as esophageal barium meal, CT, MRI, etc. at the end of treatment.

### Follow-up and statistical analysis

The patients follow-up visits were conducted one month after treatment completion, every 3 months in the first 2 years, and 6 months thereafter. All statistical analyses were performed using the SPSS 22.0 software package (IBM Corp., Armonk, NY, USA). OS, PFS and LC were assessed using the Kaplan–Meier method, and differences between the groups were assessed using the univariate analysis of the COX regression model.

According to the following literature [16], the disease control rate (DCR) of nimotuzumab combined with concurrent chemoradiotherapy in the treatment of local advanced esophageal cancer was 79.7%, and the expected DCR in this study was 97.1%. The first class error was set as unilateral 0.025, and the confidence was 80%. The Test for one proportion model in SPSS 15.0 software was used for calculation. 29 subjects were required, and 35 patients were required considering 15% shedding rate.

## Results

### Patient characteristics and treatment compliance

From December 2018 to August 2021, a total of 35 patients with stage II -IVA esophageal squamous cell cancer (ESCC) were enrolled. The mean age was 68 (range, 56 ~ 78) years, the mean lesion length measured by barium meal was 5.1 cm (range, 2 ~ 11.5 cm, two patients were unable to show the length of the lesion due to complete obstruction). Six patients opted for consolidation chemotherapy (paclitaxel monotherapy or paclitaxel in combination with platinum) following the conclusion of the clinical trial, while one patient opted for maintenance immunotherapy. The clinical characteristics of the patients are presented in Table 1.

**Table 1:** Patient characteristics at baseline

Characteristics	Value(*n* = 35)
Mean age(years) ± SD	68 ± 7.4
Sex	
Male	21(60%)
Female	14(40%)
ECOG PS	
0 ~ 1	29(82.9%)
2	6(17.1%)
Tobacco smoking	14(40%)
Chronic obstructive pulmonary disease	5(14.3%)
Family history	17(48.6%)
Lesion length(by barium meal, cm)	5.1 ± 1.8
Tumor Location	
Cervical	2(5.7%)
Upper	12(34.3%)
Middle	17(48.6%)
Lower	4(11.4%)
cT stage	
T2	4 (11.4%)
T3	24(68.6%)
T4	7(20%)
cN stage	
N0	14(40%)
N1	11(31.4%)
N2-3	10(28.6%)
cTNM stage	
II	12(34.3%)
III	14(40%)
IVA	9(25.7%)
Mean GTV ± SD(cm^3^)	47.8 ± 38.6

Abbreviations *SD* Standard deviation, *ECOG PS* Eastern Cooperative Oncology Group (ECOG) Performance Status, *T* tumor, *N* node, *M* metastasis, *GTV* gross tumor volume

One patient did not complete radiotherapy and targeted treatment due to severe radiation esophagitis secondary electrolyte disorders and hypoalbuminemia. The patient received 25 fractions of radiotherapy and only 800 mg of nimotuzumab infusion. Two other patients had their radiotherapy course interrupted for 2 weeks due to radiation esophagitis, but completed prescription dose irradiation and sufficient treatment with nimotuzumab. The overall completion rate of the treatment plan was 97.1% (34/35).

### Safety

No grade 4 to 5 adverse events occurred in the whole group. The most common treatment-related toxic reaction was radiation esophagitis, the overall incidence was 68.6% (24/35), among them, 9 cases (25.7%) and 6 cases (17.1%) developed grade 2 and grade 3 acute esophagitis, respectively. Acute radiation pneumonitis was not common, with a total incidence rate of 8.6% (3/35), with 1 case of grade 1, 2, and 3 pneumonitis each. Other systemic adverse events included decreased blood cells, hypoproteinaemia, and electrolyte disorders. Among them, five patients experienced grade 3 electrolyte disorders during treatment (3 cases of grade 3 hyponatremia, and 2 cases of grade 3 hypokalemia). One additional patient developed a grade 2 rash. The overall occurrence of adverse events is shown in Table 2.

**Table 2:** Treatment-related acute adverse events (*n* = 35)

AE	No. of Patients(%)			
	Grade 1	Grade 2	Grade 3	Grade 4
Leucopenia	7(20%)	9(25.7%)	0	0
Neutropenia	1(2.9%)	2(5.7%)	0	0
Thrombocytopenia	0	0	0	0
Haemoglobin decreased	1(2.9%)	1(2.9%)	0	0
Transaminase abnormal	0	0	0	0
Hypoproteinaemia	21(60%)	1(2.9%)	0	0
Electrolyte disorder	11(31.4%)	2(5.7%)	5(14.3%)	0
Dyspepsia	3(8.6%)	1(2.9%)	0	0
Radiation esophagitis	9(25.7%)	9(25.7%)	6(17.1%)	**0**
Radiation pneumonitis	1(2.9%)	1(2.9%)	1(2.9%)	0
Skin rash	0	1(2.9%)	0	0

Abbreviations *AE*  Adverse event

### Efficacy

**Fig. 2:**
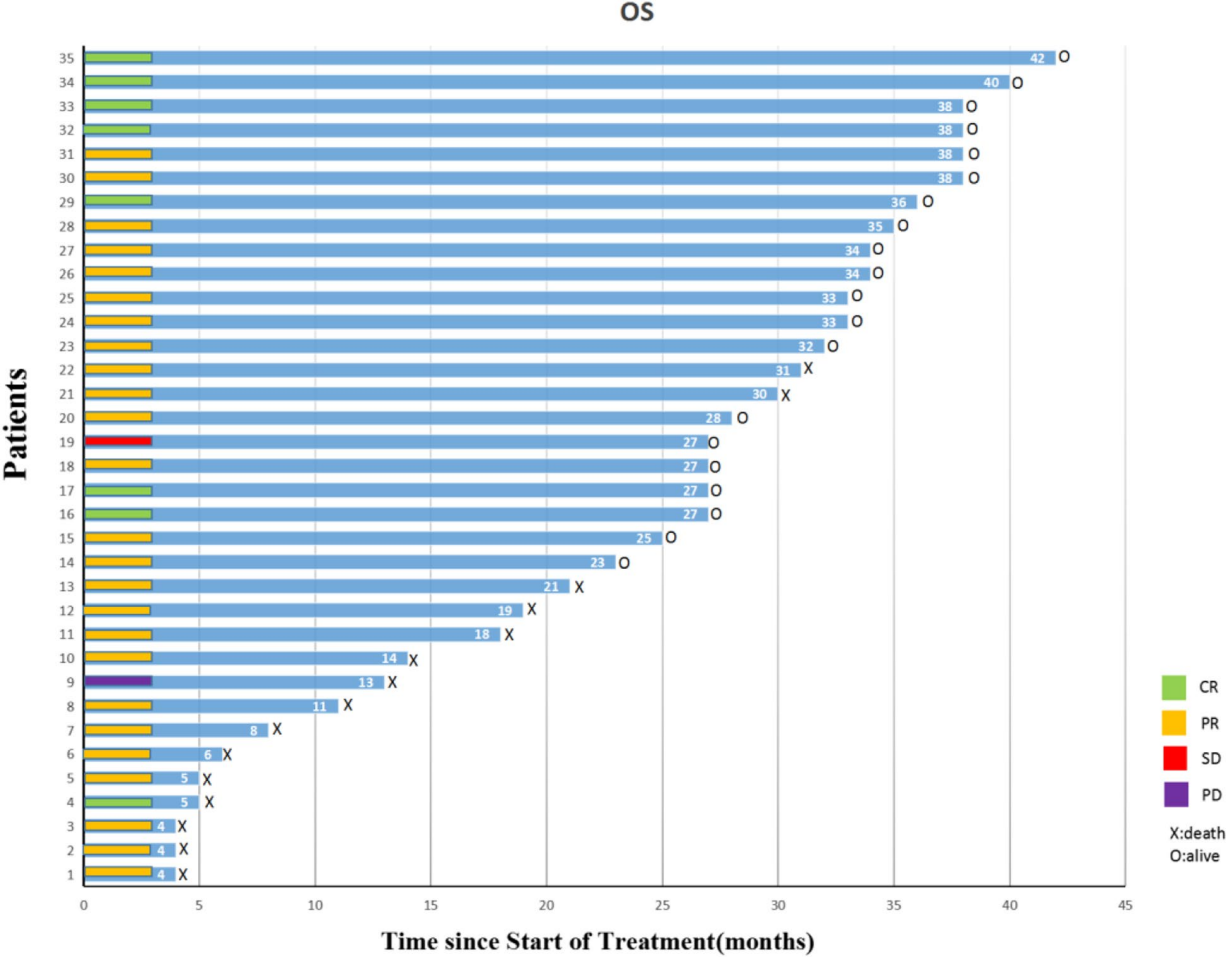
Response and survival time of ESCC patient

All 35 patients underwent therapeutic response evaluation at the end of treatment. Eight patients (22.8%) achieved complete response (CR), 25 patients (71.4%) had a partial response (PR), 1 patient (2.9%) showed stable disease (SD) and 1 case (2.9%) suffered progressive disease (PD) due to liver metastasis indicated by thoracic and abdominal enhanced CT scan. The objective response rate (ORR) was 94.2% and the disease control rate (DCR) was 97.1%. Figure 2 shows the clinical response to treatment of all patients in the study.

### Local control and survival status

**Fig. 3:**
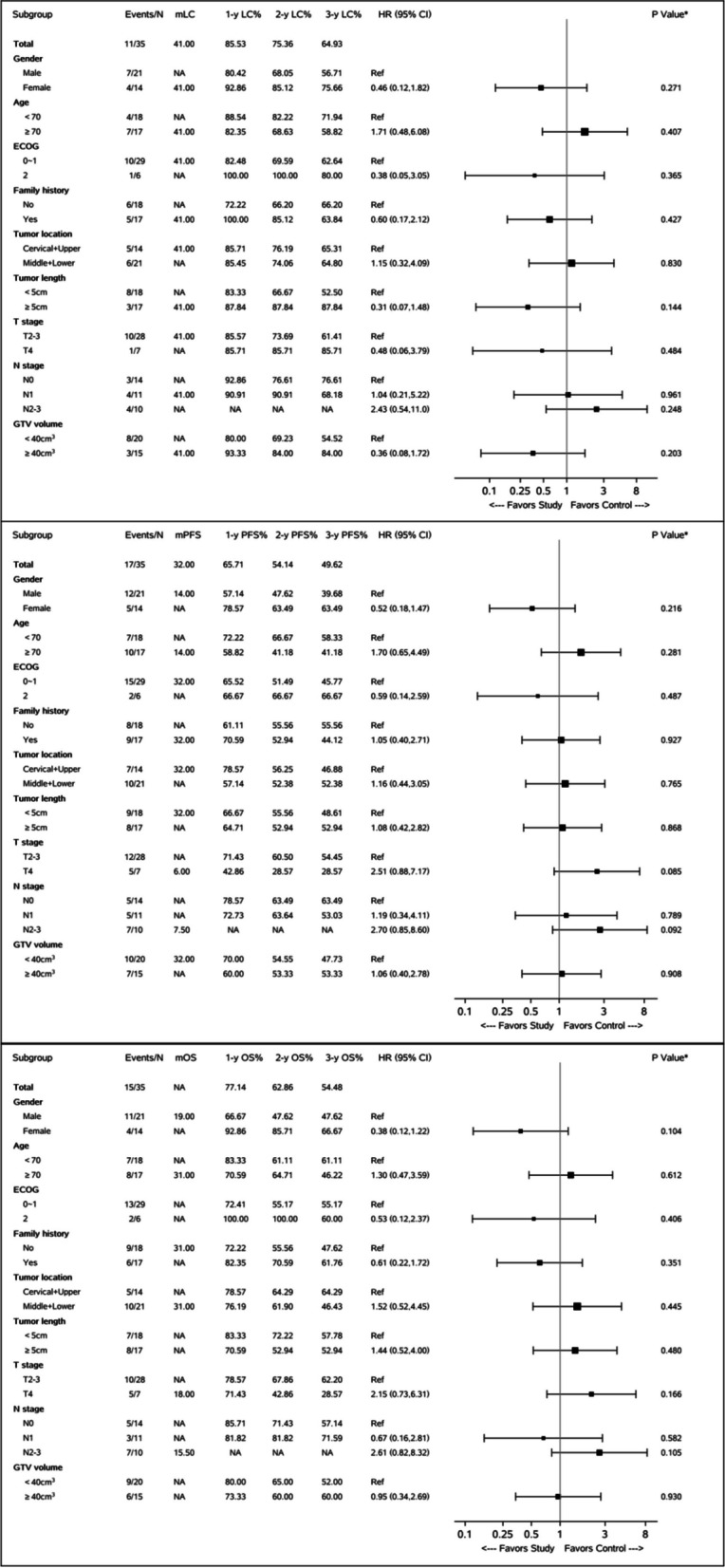
Forest pot of locally advanced unresectable esophageal cancer patient

The last follow-up date was July 24, 2023. The median follow-up time was 27 (range, 4 ~ 42) months. At the time of follow-up, 20 patients were still alive. The recurrence pattern was defined as the initial recurrence pattern during the follow-up, of the 17 patients with disease progression, 9 (52.9%) had local progression, 1(5.9%) had abdominal lymph node metastasis, 6 (35.3%) developed distant metastasis, and 1 patient (5.9%) showed both regional and distant failure. In cases of distant metastasis, the most common was lung metastasis (4 cases), followed by liver metastasis and bone metastasis. The local control and survival status of the entire group and each subgroup are shown in Fig. 3. According to the Kaplan–Meier method, the median local control time of the whole group was 35 months, nearly half (49.6%) of the patients maintained disease progression-free survival at 3 years after treatment. The 1-, 2- and 3-year overall survival rates were 77.1%, 62.9% and 54.5% respectively, and the median survival time was not reached.

In the subgroup analysis, for the subgroups of aging (≥ 70 years), poor general condition (ECOG PS = 2), longer tumor length (≥ 5 cm), and larger GTV volumes (≥ 40 cm^3^), SIB-IMRT combined with nimotuzumab could achieve similar LC, PFS and OS outcome to those of patients with younger age(< 70 year), better general conditional(ECOG PS = 0–1), shorter tumor length(< 5 cm) and smaller GTV volumes (< 40 cm^3^).

## Discussion

For locally advanced unresectable esophageal cancer, CCRT is a standard treatment option based on the landmark study of RTOG8501 [1, 2] and RTOG9405 [3] trials. Over the years, with the continuous updating and progress of radiotherapy technology equipment and chemotherapy drugs, the efficacy data of CCRT for esophageal cancer have also been improving, and the 5-year OS rate has increased from the previously reported 26% [2] to 39.9% [4]. After entering the era of immunotherapy, although multiple studies [5–9] are underway on the combination of chemoradiotherapy and immunotherapy for locally advanced esophageal cancer, however, based on the existing results, CCRT remains the most recommended treatment mode. However, in the real world, a considerable number of esophageal cancer patients were unable to receive CCRT at the time of diagnosis due to factors such as older age, malnutrition, poor performance status, severe comorbidities, and technical limitations of treatment institutions, etc. In a multi-center study of 3JECROG in China [10], only 1808 of 3977 patients (45.5%) received radial CCRT, while the rest mainly received sequential chemoradiotherapy or radiotherapy alone. The median survival time also decreased from 23.5 months to 17.6 months and 20.7 months. Therefore, for esophageal cancer patients who cannot tolerate or refuse CCRT, it is an urgent clinical problem to find a comprehensive treatment regimen with superior efficacy and low toxicity to bring therapeutic benefits to more patients.

Although under the condition of CCRT, further increasing the radiation dose from 50 to 50.4 Gy doses not seem to bring further survival benefits to patients [17, 18]. However, in the absence of sufficient systemic treatment support, the local control effect of radiotherapy seems to deserve a higher weight in the consideration scope. In the study of 3JECROG [10], we could also observe that in the condition regardless of treatment mode, the median survival times of the different irradiation dose groups of ≥ 60 Gy, 50-59 Gy, and 40—49.9 Gy were 23.3 months, 15.8 months, and 15.0 months, respectively, with the high-dose irradiation group having a survival advantage. In previous reports and our own research [11, 19], SIB-IMRT has been identified as an advantageous radiotherapy technique, capable of ensuring high-dose irradiation of the GTV region while also effectively reducing the radiation dose to normal tissues. Consequently, we have opted for an irradiation regimen consisting of a PTV-C prescription dose of 50.4 Gy, with the GTV region simultaneous integrated boost to 63 Gy, to ensure the patient's local regional control. Additionally, as a humanized anti-EGFR monoclonal antibody, nimotuzumab has demonstrated promising safety profiles and potential therapeutic benefits in prior esophageal cancer studies. For instance, in the NICE trial in Brazil [20], the treatment of locally advanced esophageal cancer using the combination of nimotuzumab and CCRT was evaluated. The results showed that the pathological complete response rate was significantly better than that of CCRT alone (62.3% vs. 37.0%, *P* = 0.02), with a trend towards an extension in the median overall survival (15.9 months vs. 11.5 months, HR = 0.68, 95%CI 0.44–1.07, *P* = 0.09). Furthermore, this treatment method did not significantly affect the quality of life for patients. Similarly, in a recently published phase III clinical trial [12], the treatment of locally advanced esophagus using nimotuzumab in combination with CCRT did not significantly increase the 3 ~ 5 grade adverse events (11.1% vs. 10.9%, *P* > 0.05), achieving an ORR rate of 93.8% and a DCR rate of 98.8%. In light of the aforementioned background, we designed this phase II clinical trial. The primary endpoint of the study is to assess the feasibility, safety, and efficacy of the novel combined treatment modality of SIB-IMRT in conjunction with nimotuzumab for locally advanced esophageal cancer. The secondary endpoint encompasses the local control and survival outcomes of the patients.

According to the research outcomes, the treatment process for all patients in the group proceeded smoothly, with only one patient failing to complete the full course of radiotherapy and nimotuzumab infusion. The overall completion rate of the research protocol is high (97.1%), indicating its feasibility. Based on the incidence of adverse reactions during the treatment, no grade 4–5 adverse events were observed in the entire group. The most common and clinically concerning toxic reaction was acute radiation esophagitis, with an overall incidence of 68.6%. Among these, the incidences of grade 2 and 3 acute esophagitis were 25.7% (9/35) and 17.1% (6/35), respectively. We attribute the significant esophagitis reactions to the high-intensity localized treatment of the lesions by the SIB-IMRT technique (total dose of 63 Gy, with a single fraction of 2.25 Gy). In previous studies on SIB-IMRT of esophageal cancer, it was similarly demonstrated that esophagitis is the primary adverse reaction associated with the radiotherapy technique. Depending on the single fraction dose of the integrated boost region, the incidence of grade 3 acute esophagitis ranges from 13 to 40% [19, 21–23]. Compared to previous data, the incidence of esophagitis in this study is comparable and falls within the clinically manageable range. Out of the six patients, five (83.3%) underwent aggressive supportive treatment and ultimately completed the full course of treatment at the recommended dosage.

In the efficacy observation, the CR rate obtained from the study was 22.8%, the PR rate was 71.4%, the ORR rate of the protocol was 94.2%, and the DCR rate was 97.1%. Only one patient was identified as PD due to the discovery of liver metastasis at the end of treatment. Recently, Xue Meng and colleagues [12] conducted an interim analysis of a phase III multi-center clinical trial of the combination of CCRT with nimotuzumab for the treatment of locally advanced unresectable esophageal cancer. In this study, the CR rate for the combined group was 32.5%, the ORR rate was 93.8%, and the DCR rate was 98.8%. Compared to data from multi-center studies, the efficacy data obtained in this study is similar and has reached a satisfactory clinical endpoint.

Based on the follow-up data on local control and survival, the 1- and 3-year local control (LC) rates, progression-free survival(PFS) rates, and overall survival(OS) rates for the entire group are 85.5% and 64.9%, 65.7% and 49.6%, 77.1% and 54.5%, respectively. Comparison of historical data on CCRT for esophageal cancer, it can be observed that in the phase III multi-center study by Yujin Xu and colleagues [17], the 1- and 3-year LC rate, PFS rate and OS rate for CCRT of 60 Gy group were 75.6% and 49.5%, 71.2% and 46.4%, 83.7% and 53.1%, respectively. For the 50 Gy group, the 1- and 3-year LC rate, PFS rate and OS rate were 72.1% and 48.4%, 65.2% and 46.1%, 84.8% and 52.7%, respectively. In the ARTDECO study [18], the 3-year OS rate for patients undergoing SIB-IMRT combined with concurrent chemotherapy was only 42% (SD group) and 39% (HD group). It appears that the treatment of locally advanced esophageal cancer with SIB-IMRT in combination with nimotuzumab can achieve local control and survival outcomes that are non-inferior to those of CCRT. Additionally, two studies from the Cancer Hospital, Chinese Academy of Medical Sciences and Peking Union Medical College [24, 25] have also explored the use of nimotuzumab in combination with radiotherapy or CCRT for the treatment of elderly patients(≥ 70 years) with esophageal cancer. In which, the 3-year OS rate for the treatment of elderly patients with esophageal cancer using the combination of nimotuzumab and radiotherapy was 21.7%, with a 3-year PFS rate of 19.6%. The median OS time for the treatment of elderly patients with esophageal cancer using nimotuzumab in combination with CCRT was 48.4 months. Both studies concluded that the treatment of esophageal cancer using nimotuzumab in combination with radiotherapy/chemoradiotherapy is safe and effective.

In summary, we believe that for locally advanced esophageal cancer patients who cannot tolerate or refuse CCRT, the treatment of SIB-IMRT combined with nimotuzumab demonstrates good feasibility and efficacy, with potential benefits in local control and survival. Acute radiation esophagitis is the primary treatment-related toxicity, but it is clinically manageable. This comprehensive treatment approach warrants further clinical exploration.

## Data Availability

The datasets used and/or analysed during the current study are available from the corresponding author on reasonable request.

## Abbreviations

AE: Adverse event
CCRT: Concurrent chemoradiotherapy
CR: Complete response
CT: Computed Tomography
CTV: Clinical tumor volume
DCR: Disease Control Rate
ECT: Emission Computed Tomography
ECOG PS: Eastern Cooperative Oncology Group Performance Status
ESCC: Esophageal squamous cell carcinoma
EUS: Endoscopic ultrasonography
GTV: Gross tumor volume
IFI: Involved-field radiotherapy
LC: Local control
MRI: Magnetic Resonance Imaging
OAR: Organs at risk
ORR: Objective response rate
OS: Overall survival
PD: Progressive disease
PET: Positron emission tomography
PFS: Progression-free survival
PR: Partial Response
PTV: Planning target volume
SD: Stable Disease
SD: Standard deviation
SIB-IMRT: Simultaneous integrated boost intensity-modulated radiotherapy

## References

[1] Herskovic A, Martz K, al-Sarraf M (1992). Combined chemotherapy and radiotherapy compared with radiotherapy alone in patients with cancer of the esophagus. N Engl J Med.

[2] Cooper JS, Guo MD, Herskovic A (1999). Chemoradiotherapy of locally advanced esophageal cancer: long-term follow-up of a prospective randomized trial (RTOG 85–01). Radiation therapy oncology group. JAMA.

[3] Minsky BD, Pajak TF, Ginsberg RJ (2002). INT 0123 (Radiation Therapy Oncology Group 94–05) phase III trial of combined-modality therapy for esophageal cancer: high-dose versus standard-dose radiation therapy. J Clin Oncol.

[4] Wang L, Wang X, Ren X (2021). Age plays an important role in the decision of definitive concurrent chemoradiotherapy (CCRT) for esophageal squamous cell carcinoma (ESCC): a propensity-score matched analysis of multicenter data (3JECROG R-02A). Transl Cancer Res.

[5] Shah MA, Bennouna J, Doi T (2021). KEYNOTE-975 study design: a phase III study of definitive chemoradiotherapy plus pembrolizumab in patients with esophageal carcinoma. Future Oncol.

[6] Wang W, Li J, Li T, et al. A phaseIII trial in progress comparing tislelizumab plus concurrent chemoradiotherapy (cCRT) with placebo plus cCRT in patients with localized esophageal squamous cell carcinoma (ESCC). J Clin Oncol. 2020;38. 10.1200/jco.2020.38.4_suppl.tps475.

[7] Wang L, Chen M, Kato K, et al. A phase 3 randomized, double-blind, placebo-controlled, multicenter, global study of durvalumab with and after chemoradiotherapy in patients with locally advanced, unresectable esophageal squamous cell carcinoma: KUNLUN. J Clin Oncol. 2022;40. 10.1200/JCO.2022.40.4_suppl.TPS373.

[8] Zhang W, Yan C, Zhang T (2021). Addition of camrelizumab to docetaxel, cisplatin, and radiation therapy in patients with locally advanced esophageal squamous cell carcinoma: a phase 1b study. Oncoimmunology.

[9] Bando H, Kotani D, Tsushima T (2020). TENERGY: multicenter phase II study of Atezolizumab monotherapy following definitive chemoradiotherapy with 5-FU plus Cisplatin in patients with unresectable locally advanced esophageal squamous cell carcinoma. BMC Cancer.

[10] Li C, Wang X, Wang L (2021). Clinical practice and outcome of radiotherapy for advanced esophageal squamous cell carcinoma between 2002 and 2018 in China: the multi-center 3JECROG Survey. Acta Oncol.

[11] Lan W, Lihong L, Chun H (2022). Comparison of efficacy and safety between simultaneous integrated boost intensity-modulated radiotherapy and standard-dose intensity-modulated radiotherapy in locally advanced esophageal squamous cell carcinoma: a retrospective study. Strahlenther Onkol.

[12] Meng X, Zheng A, Wang J (2023). Nimotuzumab plus concurrent chemo-radiotherapy in unresectable locally advanced oesophageal squamous cell carcinoma (ESCC): interim analysis from a Phase 3 clinical trial. Br J Cancer.

[13] Lu M, Wang X, Shen L (2016). Nimotuzumab plus paclitaxel and cisplatin as the first line treatment for advanced esophageal squamous cell cancer: A single centre prospective phase II trial. Cancer Sci.

[14] Park SY, Kim DJ, Suh JW (2018). Comparison of the 11th Japanese classification and the AJCC 7th and 8th staging systems in esophageal squamous cell carcinoma patients. J Thorac Dis.

[15] Eisenhauer EA, Therasse P, Bogaerts J (2009). New response evaluation criteria in solid tumours: revised RECIST guideline (version 1.1). Eur J Cancer.

[16] Jing W, Yan W, Liu Y (2019). Slight advantages of nimotuzumab versus cetuximab plus concurrent chemoradiotherapy in locally advanced esophageal squamous cell carcinoma. Cancer Biol Ther.

[17] Xu Y, Dong B, Zhu W (2022). A phase III multicenter randomized clinical trial of 60 Gy versus 50 Gy radiation dose in concurrent chemoradiotherapy for inoperable esophageal squamous cell carcinoma. Clin Cancer Res.

[18] Hulshof MCCM, Geijsen ED, Rozema T (2021). Randomized study on dose escalation in definitive chemoradiation for patients with locally advanced esophageal cancer (ARTDECO Study). J Clin Oncol.

[19] Chen D, Menon H, Verma V (2019). Results of a phase 1/2 trial of chemoradiotherapy with simultaneous integrated boost of radiotherapy dose in unresectable locally advanced esophageal cancer. JAMA Oncol.

[20] de Castro JG, Segalla JG, de Azevedo SJ (2018). A randomised phase II study of chemoradiotherapy with or without nimotuzumab in locally advanced oesophageal cancer: NICE trial. Eur J Cancer.

[21] Yu WW, Zhu ZF, Fu XL (2014). Simultaneous integrated boost intensity-modulated radiotherapy in esophageal carcinoma: early results of a phase II study. Strahlenther Onkol.

[22] Xu Y, Wang Z, Liu G (2016). The efficacy and safety of simultaneous integrated boost intensity-modulated radiation therapy for esophageal squamous cell carcinoma in Chinese population: A single institution experience. J Cancer Res Ther.

[23] Yu W, Cai XW, Liu Q (2015). Safety of dose escalation by simultaneous integrated boosting radiation dose within the primary tumor guided by (18)FDG-PET/CT for esophageal cancer. Radiother Oncol.

[24] Yu N, Cheng G, Li J (2023). Efficacy and safety of concurrent chemoradiotherapy combined with nimotuzumab in elderly patients with esophageal squamous cell carcinoma: a prospective real-world pragmatic study. Curr Cancer Drug Targets.

[25] Yang X, Zhai Y, Bi N (2021). Radiotherapy combined with nimotuzumab for elderly esophageal cancer patients: A phase II clinical trial. Chin J Cancer Res.

